# Causal effects of systemic lupus erythematosus on endometrial cancer: A univariable and multivariable Mendelian randomization study

**DOI:** 10.3389/fonc.2022.930243

**Published:** 2022-10-03

**Authors:** An Wan, Wei-Dong Zhao, Jin-Hui Tao

**Affiliations:** ^1^ Department of Rheumatology and Immunology, The First Affiliated Hospital of University of Science and Technology of China (USTC), Division of Life Sciences and Medicine, University of Science and Technology of China, Hefei, China; ^2^ Department of Obstetrics and Gynecology, The First Affiliated Hospital of University of Science and Technology of China (USTC), Division of Life Sciences and Medicine, University of Science and Technology of China, Hefei, China

**Keywords:** systemic lupus erythematosus, endometrial cancer, multivariable Mendelian randomization study, causal relationship, genetics

## Abstract

**Objective:**

Systemic lupus erythematosus (SLE) has been observationally associated with endometrial cancer, but the causality remains unclear. Here, we investigated for the first time the causal links between SLE and endometrial cancer risk.

**Methods:**

Univariable and multivariable Mendelian randomization (MR) analyses were conducted to disentangle the causality of SLE with endometrial cancer. Apart from the inverse-variance weighted (IVW) method as the primary MR estimate, three complementary MR techniques including weighted median, weighted mode, and MR-Egger regression in univariable MR were conducted to clarify the robustness of the causal estimate and mediation effects of the body mass index (BMI) and were investigated within multivariable MR-IVW and MR-Egger analyses.

**Results:**

All univariable MR analyses consistently suggested that SLE has a protective effect on the risk of overall endometrial cancer (IVW: OR = 0.956, 95% CI = 0.932-0.981, *P* = 0.001) and endometrioid endometrial cancer (IVW: OR = 0.965, 95% CI = 0.933-0.999, *P* = 0.043). More compelling, after adjustment for BMI within the multivariable MR setting, the association between SLE and decreased risk of overall endometrial cancer was significantly stronger (IVW: OR = 0.952, 95% CI = 0.931-0.973, *P* = 9.58E-06).

**Conclusions:**

Our findings provide evidence of a significant causal relationship between SLE and decreased endometrial cancer risk. Further understanding of the underlying mechanisms linking SLE with endometrial cancer is therefore needed.

## Introduction

Systemic lupus erythematosus (SLE), a multisystem, potentially fatal autoimmune disease, is characterized by the immune system attacking healthy cells and tissues throughout the body (1, 2). Because advances in treatment have improved life expectancy for patients with SLE, comorbidities, such as malignancies, have been the focus of much attention (3). Endometrial cancer is the most commonly occurring gynecologic cancer in developed countries, with 417,367 new diagnoses made globally in 2020 (4, 5). Opposite to most cancers, the incidence of endometrial cancer has increased in recent years, especially in women under the age of 40 years, and is predicted to continue to rise during the next 10 years (6). Given that SLE is a disease predominantly of women (female: male ratio of 9:1) and the rapidly increasing incidence of endometrial cancer, a better understanding of the relationship between SLE and endometrial cancer is particularly salient. Although there are inconsistent findings, a substantial body of observational studies generally supported the hypothesis that SLE may protect against the risk of endometrial cancer (7–11). Similarly, a systematic review and meta-analysis also suggested that patients with SLE have a lower risk of endometrial cancer (8, 11). Nonetheless, traditional epidemiological studies are subject to confounding and reverse causation, which makes causal inferences difficult (12). Thus, any causal relationship between SLE and the risk of endometrial cancer remains unclear.

Mendelian randomization (MR), mimicking the design of randomized controlled trials, in particular, when RCTs are impractical or unethical, utilizes genetic determinants of the exposure to understand the causal effect of the exposure on the outcome (13, 14). Because MR relies solely on genetic elements that are randomly assigned at meiosis and remain constant over the life span of an individual; it effectively mitigates the bias affecting observational epidemiologic research (12). Recent advances in MR approaches such as weighted median MR and MR-Egger enabled us to assess the robustness of the causal estimates in the presence of pleiotropy (15).

In the absence of RCTs, this study, therefore, leveraged univariable MR and multivariable MR approaches to disentangle the potential causal relationship of SLE with the risk of endometrial cancer in a two-sample MR design.

## Methods

### Study design and data source

**Figure 1:**
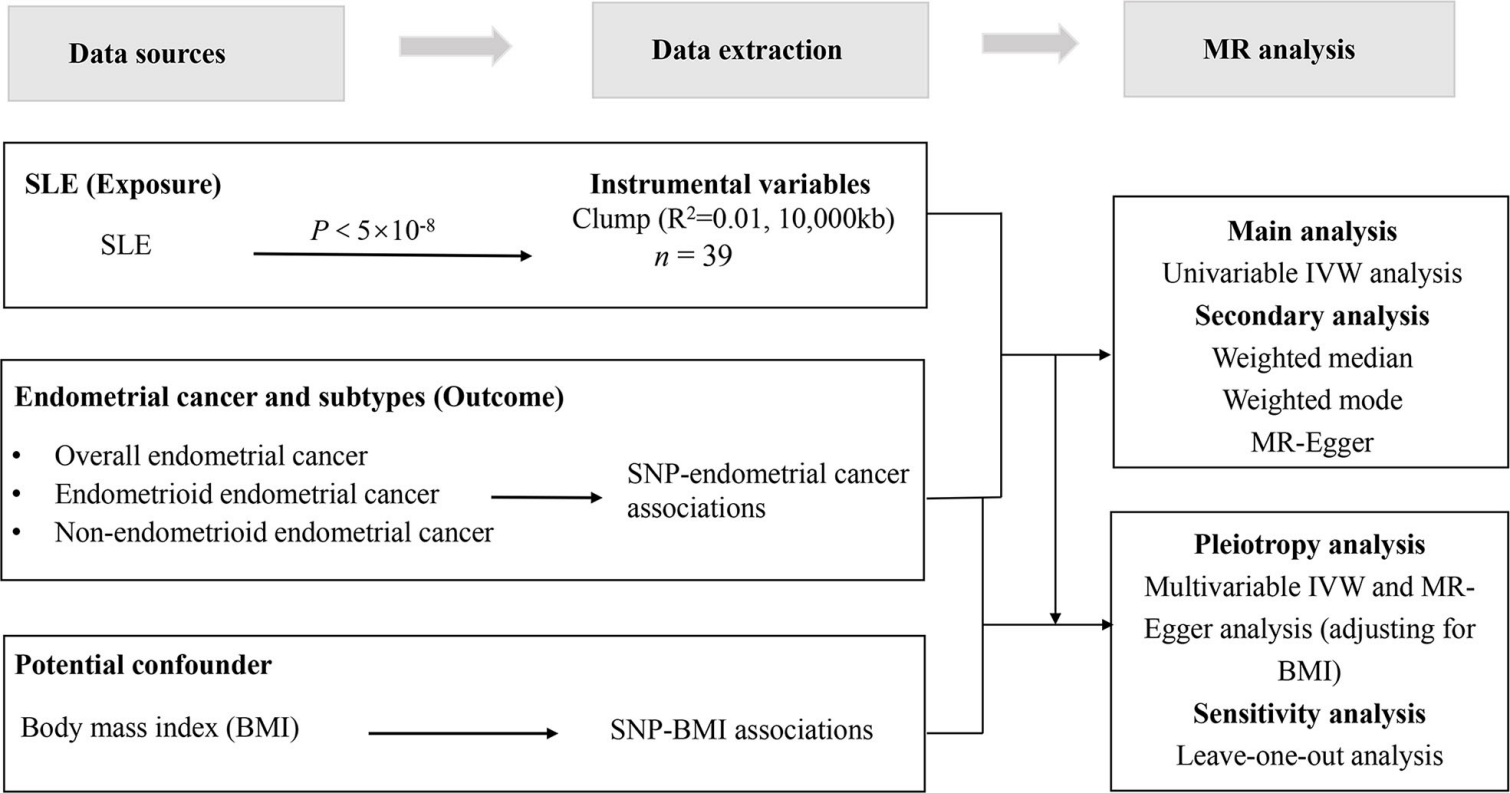
The overview design of the present study. MR, Mendelian randomization. SNP, single-nucleotide polymorphism.

A full description of the research design is elaborated in **Figure 1**. By leveraging univariable and multivariable two-sample MR analyses, our study appraised the causal impact of SLE on endometrial cancer and its subtypes (endometrioid and non-endometrioid endometrial cancer). Genetic association estimates for SLE were acquired from the largest currently available GWAS repository on SLE (*n* = 23,210), which included a new GWAS (*n* = 10,995), a meta-analysis with a published GWAS (*n* = 3,272), and a replication study (*n* = 8,943), involving a total of 7,219 SLE cases and 15,991 controls (16). For endometrial cancer, the summary-level statistics of a meta-analysis of GWAS conducted by O’Mara et al. (17) comprising 12,906 cases and 108,979 country-matched controls from 17 studies were considered. Meanwhile, endometrioid (8,758 cases and 46,126 controls) and non-endometrioid (1,230 cases and 35,447 controls) endometrial cancers are subsets of the GWAS meta-analysis of endometrial cancer based on histological subtype (17). To minimize potential bias from population stratification, we restricted the data for SLE and endometrial cancer to European-descent individuals only. In addition, we also performed multivariable MR to test the mediation effect of BMI (one of the major risk factors for endometrial cancer), which was correlated with SLE (18, 19). Publicly available genetic data for BMI were retrieved from the most recent GWAS meta-analysis, comprising 694,649 participants of European ancestry (20). **Supplementary Table 1** describes in detail the contributing studies. No ethics approval was required as all analyses in our study utilize publicly available summary data.

### Selection of the genetic instruments

In both univariable and multivariable MR analyses, the employed instrumental variables (IVs) were the same, and they all met the MR assumptions (**Supplementary Figure 1**). Specifically, SNPs associated with the exposure (here, SLE) at the genome-wide significance threshold (*P<* 5 × 10^-8^) were initially selected as the IVs for MR analyses. We then pruned them for linkage disequilibrium (*R* ^2^< 0.01 at a 10,000-kb window) to ensure that they were independent of each other. From the dataset of outcome (here, overall endometrial cancer, endometrioid and non-endometrioid endometrial cancer), we extracted the summary statistics of these selected genetic instruments. For genetic variants that were not ascertained in the GWAS repository on endometrial cancer, we identified a proxy variant with strong LD (*R* ^2^ > 0.8 in European ancestry samples from the 1,000 Genomes Project) and present in the endometrial cancer datasets to substitute it but removed it from our MR analysis if no such proxy could be found. In addition, potential pleiotropic genetic variants that showed a suggestive association (*P*< 1 × 10^-5^) with endometrial cancer and SNPs for being palindromic with intermediate frequencies were removed. Furthermore, we then derived the *R* ^2^ of each SNP and summed them up to the computer F-statistic to test for weak instrument bias with the following equation: *F*-*statistic* =*R*²×(*SampleSize*−2)/(1−*R*²) (21).

### Univariable Mendelian analysis

The random-effect inverse variance-weighted (IVW) MR was applied as the primary method, which uses the reciprocal of the outcome variance as weight for fitting and provides the most precise causal estimation if all IVs are valid (22, 23). Three complementary MR methods including weighted-median (24), weighted mode (25), and MR-Egger (26) approaches, which make weaker IV assumptions than the standard IVW approach at the cost of reduced statistical power, were conducted to clarify the robustness of the IVW estimate. In general, regarding efficiency, the estimates from the weighted-median method, which may come to an unbiased estimate of causality even when up to 50% of the weight comes from invalid IVs using the median instrumental variable from all IVs (24), and the weighted mode approach, which requires a number of instruments that demonstrate the same causal effect, are valid IVs and are nearly as precise as the IVW method and both are more accurate than the MR-Egger method (25). The MR-Egger approach allows free assessment of the intercept as an indicator of average pleiotropic bias and executes a weighted linear regression and gives the corrected MR estimates even when none of the instruments are valid (26).

### Multivariable Mendelian analysis

Multivariable MR analysis, an extension of univariable MR, was applied to infer the direct causal effect of SLE on endometrial cancer by keeping the potential mediator constant (27). Given that both SLE and endometrial cancer are linked to BMI, we adopted multivariable MR analysis to take into account the interrelationship between SLE and BMI. Specifically, we derived BMI-associated and SLE-associated genetic variants (*P*< 5 × 10^-8^) and then merged all genetic variants. After removing the duplicate IVs, we acquired the relevant information of each IV from the SLE, BMI, and endometrial cancer. For multivariable MR analysis, we utilized IVW with multiplicative random effects as the main analysis and MR-Egger with multiplicative random effects methods as the complementary analysis to appraise the causal effects of SLE and BMI on endometrial cancer.

### Pleiotropy and sensitivity analysis

The MR-Egger intercept test and funnel plot, which are capable of reminding the presence of horizontal pleiotropy, were applied to measure potential pleiotropy (26, 28, 29). When the intercept of the MR-Egger significantly differs from zero (*P<* 0.05) or the funnel plot asymmetry is present, horizontal pleiotropy is proposed (28, 29). In addition, leveraging the Cochran Q statistic, the heterogeneity between the causal inference across all IVs in IVW and MR-Egger approaches was examined. There was evidence of heterogeneity if the *P* value of the Cochran Q statistic was less than 0.05. Besides, two additional analyses were applied to detect SNPs reflecting pleiotropic bias: the first, by sequentially removing each SNP from the analysis, we conducted leave-one-out analysis to identify high influence points; the other, by searching the PhenoScanner v2 database for pleiotropic IVs related with the aforementioned confounder (*P<* 1 × 10^-5^), we carried out a sensitivity analysis by manually removing them (30).

### Statistical analysis

Throughout the current study, we employed the packages ‘TwoSampleMR’ (29) and ‘Mendelian Randomization’ (31) in RStudio (version 1.2.5019) to carry out all the analyses. Forest plots and funnel plots were generated using the “gglot2” package. The MR results were presented as odds ratio (OR) interpreted as endometrial cancer risk per decrease in log odds of SLE. We also reported corresponding 95% confidence intervals (CIs) lower and upper for all causal estimates. A *P*-value below 0.05 was used to define statistical significance.

## Results

### Genetic instruments

Of the 42 conditionally independent SNPs associated with SLE at the genome-wide significance threshold (*P*<5 × 10^-8^), 39 were directly available in the summary statistics data for endometrial cancer and its subtypes, of which 38 were independent from endometrial cancer and its subtypes. Additionally, one variant had a good genetic proxy (*r* ^2^ > 0.9) and was therefore added to our instrument lists, for a total of 39 variants. The F-statistic of every genetic instrument was above the threshold 10 (ranging from 32 to 754, with a mean of 123.33), thus ruling out the possibility of weak instrument bias. All 39 SNPs together accounted for 20.44% of the variance in SLE. The characteristics of all genetic instruments for SLE included in our study and their associated estimates with endometrial cancer and its subtypes are elaborated in **Supplementary Table 2**.

### Causal association of SLE with endometrial cancer *via* univariable MR

**Figure 2:**
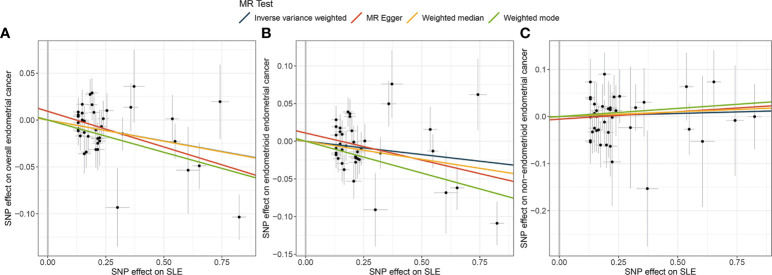
Scatter plots for effect sizes of SNPs for and those for endometrial cancer and subtypes. **(A)** Causal estimates for SLE on overall endometrial cancer. **(B)** Causal estimates for SLE on endometrioid endometrial cancer. **(C)** Causal estimates for SLE on non-endometrioid endometrial cancer. Each black point representing an SNP is plotted in relation to the effect size of the SNP on SLE (x-axis) and on the risk of endometrial cancer, endometrioid endometrial cancer and non-endometrioid endometrial cancer (y-axis) with corresponding standard error bars. The slope of each line corresponds to the causal estimate using the inverse variance weighted (blue), MR-Egger (red), weighted median (orange), and weighted mode (green) methods.

The univariable IVW method suggested a protective causal relationship between SLE and the risk of endometrial cancer (OR = 0.956, 95% CI: 0.932-0.981, *P* = 0.001). This effect was also found in histological subtypes of endometrioid endometrial cancer (IVW: OR = 0.965, 95% CI: 0.933-0.999, *P* = 0.043) but not in non-endometrioid endometrial cancer (IVW: OR = 1.031, 95% CI: 0.949-1.081, *P* = 0.693) (**Table 1** and **Figure 2**). Likewise, the results for the weighted-median, weighted mode, and MR-Egger methods were qualitatively similar to the results estimated by the IVW method (**Table 1** and **Figure 2**).

**Table 1:** Causal relationships of SLE on endometrial cancer and histological subtypes estimated by univariable MR.

Outcome	IVs	OR	95% CI lower	95% CI upper	P	Method
Overall endometrial cancer	39	0.956	0.932	0.981	0.001	IVW
	39	0.956	0.921	0.992	0.018	Weighted median
	39	0.934	0.883	0.987	0.021	Weighted mode
	39	0.927	0.883	0.972	0.003	MR-Egger
Endometrioid endometrial cancer	39	0.965	0.933	0.999	0.043	IVW
	39	0.953	0.912	0.996	0.033	Weighted median
	39	0.919	0.870	0.971	0.004	Weighted mode
	39	0.931	0.872	0.993	0.037	MR-Egger
Non-endometrioid endometrial cancer	39	1.013	0.949	1.081	0.693	IVW
	39	1.020	0.926	1.124	0.684	Weighted median
	39	1.035	0.934	1.147	0.512	Weighted mode
	39	1.032	0.913	1.167	0.616	MR-Egger

Although the testing of Cochran’s Q test revealed evidence of heterogeneity, the MR-Egger intercept test and the symmetry in funnel plots implied the absence of horizontal pleiotropy (**Supplementary Table 3** and **Supplementary Figure 2**). Similarly, leave-one-out analysis (**Supplementary Table 4**) and forest plots (**Supplementary Figure 3**)did not detect any leverage points with high influence. In the PhenoScanner database, we identified three (rs9462027, rs849142, and rs2736340) of the 39 SNPs for SLE associated with body mass index. However, removing these three SNPs did not change the pattern of results (**Supplementary Table 5**).

### Causal association of SLE with endometrial cancer *via* multivariable MR

After adjustment for BMI within the multivariable MR setting, the association between SLE and decreased risk of overall endometrial cancer was significantly stronger (IVW: OR = 0.952, 95% CI: 0.931-0.973, *P* = 9.58E-06). Beyond that, the effect of SLE on endometrioid endometrial cancer risk lost the statistical significance after adjustment for BMI (OR = 0.993, 95% CI: 0.970-1.015, *P* = 0.525). The multivariable MR-Egger regression supported largely these findings (overall endometrial cancer: OR = 0.949, 95% CI: 0.926-0.972, *P* = 2.47E-05; endometrioid endometrial cancer: OR = 0.993, 95% CI: 0.966-1.020, *P* = 0.594). Moreover, an insignificant multivariable MR-Egger intercept implied the absence of horizontal pleiotropy (all *P*_inter_ > 0.05), indicating the robustness of the multivariable MR analysis results.

## Discussion

This is the first MR study to quantify the potential causality between SLE and the risk of endometrial cancer and its subtypes by performing univariate and multivariate MR analyses, and we found evidence supporting a potential causal relationship between SLE and the reduced risk of endometrial cancer. Strikingly, the results were robust in a series of sensitivity analyses.

Previously, epidemiological observations have extensively reported a strong association of SLE with decreased risk of endometrial cancer, although there are conflicting results. For instance, an international, multisite (30 centers), prospective, cohort study, comprising 16,409 SLE patients observed for 121,283 patient years, demonstrated that SLE patients have a decreased risk of endometrial cancer (standardized incidence ratio (SIR) = 0.44, 95% CI: 0.23-0.77) (7). In addition, a meta-analysis of five large SLE cohorts involving 47,325 SLE patients and observing for a total of 282,553 person-years strongly supported a protective role of SLE for endometrial cancer (SIR = 0.71, 95% CI: 0.55-0.91) (11). Furthermore, a systematic review corroborated the results from these aforementioned studies which showed that SLE was associated with decreased risk of endometrial cancer (8). By leveraging a genetic approach, the current study added to the growing body of data that SLE lowered the risk of endometrial cancer.

Multivariable MR analysis takes pleiotropy among multiple traits into account and assesses the causal effect of numerous exposure variables on an outcome (32, 33). More specifically, by keeping the potential mediator (secondary exposure) constant, this approach enabled us to assess separate but related exposures simultaneously and generated a more reliable identification of a causal association between exposure (main exposure) and outcome (14). More compelling, in our findings, the association between SLE and decreased risk of overall endometrial cancer was significantly stronger, after adjustment for BMI.

Although the biological mechanism of how SLE lowered the risk of endometrial cancer remains unclear, there are several plausible explanations. For example, studies have suggested that SLE patients had significantly higher levels of sex hormone binding globulin (SHBG) in comparison with the controls, resulting in decreased concentrations of bioavailable estrogen (the most potent estrogen receptor agonist), which may reduce the risk of endometrioid endometrial cancer (34). In addition, autoantibodies reactive against host DNA were observed in the circulation of SLE patients, and their aberrant production is a hallmark of SLE (35–37). A recent study reported that a cell-penetrating lupus autoantibody, 3E10, is highly and selectively toxic to cancer cells and tumors with defective homology-directed repair of DNA double-strand breaks (38). Mechanistically, 3E10 can bind DNA single-strand tails, resulting in the inhibition of key steps in DNA single-strand and double-strand break repair (37, 38). The aforementioned findings indicate that the protective role of SLE for endometrial cancer may be related to the presence of certain nuclear-penetrating autoantibodies that are particularly lethal to cancer cells with preexisting defects in DNA repair.

This MR study has several strengths. The MR design is a major strength, allowing us to infer causality between two diseases, which is infeasible by randomized clinical trials (RCTs) (39). Leveraging multiple MR analyses, especially multivariable MR approaches that account for potential confounding owing to BMI, we clarified the association between genetic liability for SLE and endometrial cancer. Another strength is the application of the largest possible and well-powered GWAS for SLE and endometrial cancer, which strengthened the power and provided a possibility to examine the links between endometrial cancer subtypes. Notwithstanding, the current MR study still has some limitations. First, all participants were of European ancestry, thus making our findings potentially inadequate for generalizing to populations of other ethnicities, while eliminating the bias due to ethnic heterogeneity. Second, due to limited information, the degree of sample overlap between SLE and endometrial cancer datasets could not be assessed, potentially biasing the estimates toward the observational correlation in the presence of a weak instrument (40). However, weak instrument bias is not expected given that the SNPs from the SLE GWAS are strong instruments with high F-statistic (average F-statistic = 123.33). Third, we attempted to assess the mediation effect of BMI on the relationship between SLE and endometrial cancer. However, we acknowledged that BMI is not the only potential mediator and future studies on additional potential mediators are warranted. Finally, similar to all MR studies, residual pleiotropy might remain. However, we have addressed this issue by employing a range of sensitivity analyses; therefore, the findings of the current study are unlikely to change.

Taken together, we demonstrated, for the first time utilizing univariable and multivariable MR approaches, a protective relationship between SLE and the risk of endometrial cancer. Further investigations to demystify its underlying mechanisms are needed and may open up new therapeutic avenues for endometrial cancer.

## Data availability statement

The original contributions presented in the study are included in the article/**Supplementary Material**. Further inquiries can be directed to the corresponding authors.

## Author contributions

J-HT conceived the present idea and critically revised this manuscript. W-DZ was involved in the acquisition of data and statistical analysis. AW wrote the manuscript. All authors contributed to the article and approved the submitted version.

## Funding

This study was funded by grants from the National Natural Science Foundation of China (81771774) and Anhui Provincial Key Research and Development Plan (201904a07020103).

## Conflict of interest

The authors declare that the research was conducted in the absence of any commercial or financial relationships that could be construed as a potential conflict of interest.

## Publisher’s note

All claims expressed in this article are solely those of the authors and do not necessarily represent those of their affiliated organizations, or those of the publisher, the editors and the reviewers. Any product that may be evaluated in this article, or claim that may be made by its manufacturer, is not guaranteed or endorsed by the publisher.
