# Supplementary material for: Causal effects of systemic lupus erythematosus on endometrial cancer: A univariable and multivariable Mendelian randomization study
Source: Front Oncol. 2022 Oct 3;12:930243. doi: 10.3389/fonc.2022.930243 (PMC9575983; doi:10.3389/fonc.2022.930243)
Supplement: Supplementary file 1 [file DataSheet_1.docx]

Supplementary Materials

**Table S1.** Sources of data for the analysis

| **Phenotype** | **Author, published year** | **Population** | **Participants** | **PMID** |
| --- | --- | --- | --- | --- |
| Systemic lupus erythematosus | Bentham J, et al, 2015 | European | 23,210 individuals (7,219 systemic lupus erythematosus cases and 15,991 controls) | 26502338 |
| Endometrial cancer |  |  |  |  |
| Overall endometrial cancer | O'Mara TA et al, 2018 | European | 121,885 individuals (12,906 endometrial cancer cases and 108,979 general population) | 30093612 |
| Endometrioid endometrial cancer | O'Mara TA et al, 2018 | European | 54,884 individuals (8,758 endometrial cancer cases with endometrioid histology and 46,126 general population) | 30093612 |
| Non-endometrioid endometrial cancer | O'Mara TA et al, 2018 | European | 36,677 individuals (1,230 cases with non-endometrioid histology and 35,447 general population) | 30093612 |
| Body mass index | Pulit SL et al, 2019 | European | 694,649 individuals | 30239722 |

**Table S2.** Detailed information for instrumental variables of SLE and association with endometrial cancer

| **Sort** | **SNP** | **Chr** | **Position** | **A1** | **A2** | **EAF** | **Beta** | **SE** | **P-value** | **R^2^** | **F-statistic** | **P_1_** | **P_2_** | **P_3_** |
| --- | --- | --- | --- | --- | --- | --- | --- | --- | --- | --- | --- | --- | --- | --- |
| 1 | rs10028805 | 4 | 102737250 | G | A | 0.632 | 0.182 | 0.022 | 4.31E-17 | 2.94E-03 | 68 | 9.41E-02 | 4.38E-02 | 6.53E-01 |
| 2 | rs10036748 | 5 | 150458146 | T | C | 0.250 | 0.322 | 0.023 | 1.27E-45 | 8.37E-03 | 196 | 4.03E-01 | 4.50E-01 | 7.05E-01 |
| 3 | rs10488631 | 7 | 128594183 | C | T | 0.116 | 0.652 | 0.029 | 9.37E-110 | 2.13E-02 | 505 | 4.63E-02 | 3.28E-02 | 2.73E-01 |
| 4 | rs1059312 | 12 | 129278864 | G | A | 0.388 | 0.157 | 0.021 | 1.48E-13 | 2.40E-03 | 56 | 3.98E-01 | 5.41E-01 | 9.99E-01 |
| 5 | rs11644034 | 16 | 85972612 | G | A | 0.765 | 0.223 | 0.026 | 9.58E-18 | 3.16E-03 | 74 | 2.20E-01 | 2.83E-01 | 9.75E-01 |
| 6 | rs11889341 | 2 | 191943742 | T | C | 0.226 | 0.548 | 0.023 | 5.59E-122 | 2.39E-02 | 568 | 2.25E-01 | 5.57E-01 | 6.21E-01 |
| 7 | rs1270942 | 6 | 31918860 | G | A | 0.118 | 0.824 | 0.03 | 2.25E-165 | 3.15E-02 | 754 | 1.93E-05 | 1.54E-04 | 9.97E-01 |
| 8 | rs12802200 | 11 | 566936 | C | A | 0.808 | 0.207 | 0.034 | 8.81E-10 | 1.59E-03 | 37 | 1.02E-01 | 2.33E-02 | 7.79E-01 |
| 9 | rs17849501 | 1 | 183542323 | T | C | 0.045 | 0.742 | 0.037 | 3.45E-88 | 1.70E-02 | 402 | 6.21E-01 | 1.91E-01 | 9.43E-01 |
| 10 | rs1801274 | 1 | 161479745 | G | A | 0.527 | 0.148 | 0.021 | 1.04E-12 | 2.14E-03 | 50 | 9.96E-01 | 8.42E-01 | 5.59E-01 |
| 11 | rs2111485 | 2 | 163110536 | G | A | 0.615 | 0.140 | 0.021 | 1.27E-11 | 1.91E-03 | 44 | 2.81E-01 | 2.17E-01 | 4.70E-01 |
| 12 | rs2286672 | 17 | 4712617 | T | C | 0.075 | 0.223 | 0.038 | 2.93E-09 | 1.48E-03 | 34 | 5.24E-01 | 6.88E-01 | 3.02E-01 |
| 13 | rs2289583 | 15 | 75311036 | A | C | 0.298 | 0.174 | 0.022 | 6.22E-15 | 2.69E-03 | 63 | 2.97E-01 | 7.46E-01 | 2.09E-01 |
| 14 | rs2304256 | 19 | 10475652 | C | A | 0.712 | 0.215 | 0.030 | 3.50E-13 | 2.21E-03 | 51 | 2.44E-01 | 3.06E-01 | 7.08E-01 |
| 15 | rs2431697 | 5 | 159879978 | T | C | 0.571 | 0.231 | 0.021 | 8.01E-28 | 5.19E-03 | 121 | 6.60E-01 | 1.94E-01 | 3.71E-01 |
| 16 | rs2476601 | 1 | 114377568 | A | G | 0.102 | 0.358 | 0.032 | 1.10E-28 | 5.36E-03 | 125 | 5.96E-01 | 9.73E-02 | 6.77E-01 |
| 17 | rs2663052 | 10 | 50069395 | G | A | 0.550 | 0.148 | 0.025 | 5.25E-09 | 1.51E-03 | 35 | 2.73E-01 | 3.29E-01 | 8.79E-01 |
| 18 | rs2732549 | 11 | 35088399 | A | G | 0.556 | 0.215 | 0.021 | 1.20E-23 | 4.50E-03 | 105 | 1.09E-01 | 2.49E-01 | 7.61E-01 |
| 19 | rs2736340 | 8 | 11343973 | T | C | 0.249 | 0.255 | 0.028 | 6.28E-20 | 3.56E-03 | 83 | 5.96E-01 | 9.88E-01 | 4.52E-01 |
| 20 | rs2941509 | 17 | 37921194 | T | C | 0.039 | 0.300 | 0.052 | 7.98E-09 | 1.43E-03 | 33 | 2.51E-02 | 6.23E-02 | 8.55E-01 |
| 21 | rs3024505 | 1 | 206939904 | A | G | 0.158 | 0.157 | 0.027 | 4.64E-09 | 1.45E-03 | 34 | 8.59E-02 | 2.31E-01 | 8.35E-01 |
| 22 | rs34572943 | 16 | 31272353 | A | G | 0.101 | 0.536 | 0.029 | 3.39E-76 | 1.45E-02 | 342 | 9.59E-01 | 6.01E-01 | 3.76E-01 |
| 23 | rs3768792 | 2 | 213871709 | G | A | 0.140 | 0.215 | 0.029 | 1.21E-13 | 2.36E-03 | 55 | 1.55E-01 | 2.82E-01 | 3.22E-01 |
| 24 | rs4902562 | 14 | 68731458 | A | G | 0.439 | 0.131 | 0.021 | 6.15E-10 | 1.67E-03 | 39 | 8.52E-01 | 6.00E-01 | 2.42E-01 |
| 25 | rs4917014 | 7 | 50305863 | T | G | 0.680 | 0.166 | 0.022 | 6.39E-14 | 2.45E-03 | 57 | 4.36E-02 | 5.94E-02 | 5.68E-01 |
| 26 | rs4945008* | 11 | 71221248 | A | G | 0.259 | 0.207 | 0.022 | 1.32E-20 | 3.80E-03 | 89 | 5.50E-01 | 9.58E-01 | 5.28E-01 |
| 27 | rs4948496 | 10 | 63805617 | C | T | 0.462 | 0.131 | 0.020 | 1.04E-10 | 1.85E-03 | 43 | 4.54E-01 | 3.23E-01 | 9.78E-01 |
| 28 | rs564799 | 3 | 159728987 | C | T | 0.572 | 0.131 | 0.022 | 1.54E-09 | 1.53E-03 | 35 | 5.41E-01 | 3.87E-01 | 3.58E-01 |
| 29 | rs6568431 | 6 | 106588806 | A | C | 0.396 | 0.191 | 0.025 | 5.04E-14 | 2.51E-03 | 58 | 6.73E-02 | 7.96E-02 | 4.94E-02 |
| 30 | rs6932056 | 6 | 138242437 | C | T | 0.030 | 0.604 | 0.052 | 1.97E-31 | 5.78E-03 | 135 | 2.46E-01 | 2.10E-01 | 7.04E-01 |
| 31 | rs704840 | 1 | 173226195 | G | T | 0.295 | 0.199 | 0.022 | 3.12E-19 | 3.51E-03 | 82 | 6.25E-01 | 4.81E-01 | 2.13E-01 |
| 32 | rs7444 | 22 | 21976934 | C | T | 0.200 | 0.239 | 0.025 | 1.84E-22 | 3.92E-03 | 91 | 9.39E-01 | 6.45E-01 | 8.18E-01 |
| 33 | rs7726414 | 5 | 133431834 | T | C | 0.041 | 0.372 | 0.046 | 4.44E-16 | 2.81E-03 | 65 | 3.52E-01 | 8.99E-02 | 2.13E-01 |
| 34 | rs7941765 | 11 | 128499000 | C | T | 0.516 | 0.131 | 0.020 | 1.35E-10 | 1.85E-03 | 43 | 7.19E-01 | 1.25E-01 | 2.39E-01 |
| 35 | rs849142 | 7 | 28185891 | T | C | 0.506 | 0.131 | 0.020 | 8.61E-11 | 1.85E-03 | 43 | 5.73E-01 | 2.77E-01 | 3.92E-01 |
| 36 | rs9311676 | 3 | 58470351 | C | T | 0.608 | 0.157 | 0.021 | 3.06E-14 | 2.40E-03 | 56 | 9.46E-01 | 6.27E-01 | 5.18E-01 |
| 37 | rs9462027 | 6 | 34797241 | A | G | 0.266 | 0.131 | 0.023 | 7.55E-09 | 1.40E-03 | 32 | 8.19E-01 | 6.85E-01 | 1.30E-01 |
| 38 | rs9652601 | 16 | 11174365 | G | A | 0.677 | 0.191 | 0.023 | 7.42E-17 | 2.96E-03 | 69 | 3.20E-01 | 6.03E-02 | 8.03E-01 |
| 39 | rs9782955 | 1 | 236039877 | C | T | 0.754 | 0.148 | 0.024 | 1.25E-09 | 1.64E-03 | 38 | 6.76E-01 | 5.48E-01 | 6.25E-01 |

A1, Effect allele; A2, Reference allele; EAF, allele frequency of effect allele; SE, standard error of beta; The *P* values for overall endometrial cancer (P_1_), endometrioid endometrial cancer (P_2_) and non-endometrioid endometrial cancer (P_3_); *rs4945008 (A/G) was used as proxy SNP (linkage disequilibrium R^2^=0.91) of rs3794060 (C/T) in the summary statistics of the outcome; Two SNPs (rs1734787 and rs887369) could not find good proxy replaced variant (R^2^ > 0.8) and one SNP (rs10774625) was associated with endometrial cancer and its subtypes, therefor, these three SNPs were excluded as instrumental variables for SLE.

**Table S3.** Heterogeneity tests and MR-Egger intercept of SLE causally linked to endometrial cancer

| **Outcome** | **Heterogeneity** | | | | | | **MR-Egger** | |
| --- | --- | --- | --- | --- | --- | --- | --- | --- |
|  |  | **IVW** |  |  | **MR-Egger** |  |  |  |
|  | **Cochrane Q** | **Q_df** | **P-value** | **Cochrane Q** | **Q_df** | **P-value** | **Intercept** | **P-value** |
| Overall endometrial cancer | 48.448 | 38 | 1.19E-01 | 45.669 | 37 | 1.55E-01 | 0.009 | 1.42E-01 |
| Endometrioid endometrial cancer | 63.018 | 38 | 6.56E-03 | 60.307 | 37 | 9.10E-03 | 0.011 | 2.05E-01 |
| Non-endometrioid endometrial cancer | 24.100 | 38 | 9.61E-01 | 23.978 | 37 | 9.52E-01 | -0.006 | 7.29E-01 |

**Table S4** Leave-one-out sensitivity analysis for MR analysis of the causal effect of SLE on endometrial cancer

| **Sort** | **SNP** | **OR_1_(95% CI)** | **P_1_** | **OR_2_(95% CI)** | **P_2_** | **OR_3_(95% CI)** | **P3** |
| --- | --- | --- | --- | --- | --- | --- | --- |
| 1 | rs10028805 | 0.953(0.930-0.977) | 1.28E-04 | 0.961(0.930-0.994) | 2.06E-02 | 1.011(0.947-1.080) | 7.34E-01 |
| 2 | rs10036748 | 0.956(0.931-0.982) | 8.42E-04 | 0.966(0.932-1.001) | 5.56E-02 | 1.011(0.946-1.080) | 7.48E-01 |
| 3 | rs10488631 | 0.959(0.934-0.985) | 2.27E-03 | 0.971(0.937-1.007) | 1.11E-01 | 1.002(0.935-1.073) | 9.65E-01 |
| 4 | rs1059312 | 0.957(0.932-0.982) | 7.78E-04 | 0.966(0.933-1.000) | 5.08E-02 | 1.013(0.949-1.082) | 6.91E-01 |
| 5 | rs11644034 | 0.957(0.933-0.982) | 9.13E-04 | 0.967(0.933-1.001) | 5.70E-02 | 1.014(0.949-1.082) | 6.87E-01 |
| 6 | rs11889341 | 0.956(0.930-0.982) | 1.15E-03 | 0.964(0.929-1.000) | 5.04E-02 | 1.021(0.953-1.094) | 5.54E-01 |
| 7 | rs1270942 | 0.970(0.946-0.995) | 1.86E-02 | 0.982(0.948-1.017) | 3.11E-01 | 1.016(0.946-1.090) | 6.66E-01 |
| 8 | rs12802200 | 0.958(0.933-0.982) | 9.25E-04 | 0.968(0.936-1.002) | 6.37E-02 | 1.012(0.948-1.081) | 7.16E-01 |
| 9 | rs17849501 | 0.953(0.928-0.978) | 2.47E-04 | 0.960(0.927-0.994) | 2.00E-02 | 1.014(0.949-1.084) | 6.75E-01 |
| 10 | rs1801274 | 0.956(0.931-0.981) | 5.70E-04 | 0.965(0.932-1.000) | 4.68E-02 | 1.011(0.947-1.079) | 7.39E-01 |
| 11 | rs2111485 | 0.957(0.932-0.982) | 7.94E-04 | 0.967(0.934-1.001) | 5.48E-02 | 1.016(0.952-1.084) | 6.38E-01 |
| 12 | rs2286672 | 0.956(0.932-0.981) | 7.11E-04 | 0.965(0.932-1.000) | 4.84E-02 | 1.016(0.952-1.084) | 6.33E-01 |
| 13 | rs2289583 | 0.957(0.932-0.982) | 8.27E-04 | 0.965(0.932-1.000) | 4.80E-02 | 1.018(0.954-1.087) | 5.83E-01 |
| 14 | rs2304256 | 0.957(0.933-0.982) | 9.11E-04 | 0.967(0.933-1.001) | 5.68E-02 | 1.012(0.947-1.080) | 7.31E-01 |
| 15 | rs2431697 | 0.956(0.931-0.981) | 6.67E-04 | 0.967(0.934-1.002) | 6.24E-02 | 1.008(0.944-1.077) | 8.02E-01 |
| 16 | rs2476601 | 0.954(0.930-0.979) | 3.32E-04 | 0.961(0.929-0.994) | 2.08E-02 | 1.011(0.947-1.080) | 7.40E-01 |
| 17 | rs2663052 | 0.954(0.930-0.979) | 2.75E-04 | 0.963(0.931-0.997) | 3.42E-02 | 1.013(0.949-1.081) | 7.04E-01 |
| 18 | rs2732549 | 0.958(0.933-0.983) | 1.08E-03 | 0.967(0.934-1.001) | 5.91E-02 | 1.012(0.948-1.081) | 7.24E-01 |
| 19 | rs2736340 | 0.954(0.930-0.979) | 3.44E-04 | 0.964(0.931-0.999) | 4.32E-02 | 1.010(0.945-1.078) | 7.74E-01 |
| 20 | rs2941509 | 0.958(0.934-0.982) | 6.77E-04 | 0.967(0.935-1.001) | 5.46E-02 | 1.014(0.950-1.082) | 6.82E-01 |
| 21 | rs3024505 | 0.957(0.933-0.982) | 7.57E-04 | 0.966(0.933-1.000) | 5.30E-02 | 1.013(0.949-1.081) | 7.05E-01 |
| 22 | rs34572943 | 0.953(0.929-0.979) | 3.54E-04 | 0.961(0.928-0.996) | 2.92E-02 | 1.006(0.941-1.076) | 8.57E-01 |
| 23 | rs3768792 | 0.957(0.933-0.982) | 8.59E-04 | 0.966(0.933-1.001) | 5.47E-02 | 1.017(0.953-1.086) | 6.11E-01 |
| 24 | rs4902562 | 0.956(0.931-0.981) | 6.27E-04 | 0.966(0.932-1.000) | 4.96E-02 | 1.017(0.953-1.086) | 6.08E-01 |
| 25 | rs4917014 | 0.958(0.934-0.983) | 8.76E-04 | 0.968(0.935-1.001) | 6.00E-02 | 1.015(0.951-1.084) | 6.45E-01 |
| 26 | rs4945008* | 0.956(0.932-0.981) | 7.30E-04 | 0.965(0.932-0.999) | 4.48E-02 | 1.016(0.952-1.085) | 6.31E-01 |
| 27 | rs4948496 | 0.956(0.932-0.982) | 7.39E-04 | 0.966(0.933-1.000) | 5.28E-02 | 1.013(0.949-1.082) | 6.90E-01 |
| 28 | rs564799 | 0.956(0.932-0.981) | 7.16E-04 | 0.966(0.933-1.000) | 5.21E-02 | 1.010(0.946-1.078) | 7.60E-01 |
| 29 | rs6568431 | 0.952(0.929-0.976) | 9.12E-05 | 0.961(0.929-0.994) | 2.24E-02 | 1.004(0.940-1.072) | 9.01E-01 |
| 30 | rs6932056 | 0.957(0.932-0.982) | 9.20E-04 | 0.967(0.934-1.001) | 6.00E-02 | 1.015(0.951-1.084) | 6.50E-01 |
| 31 | rs704840 | 0.954(0.930-0.979) | 3.88E-04 | 0.963(0.930-0.997) | 3.52E-02 | 1.019(0.955-1.088) | 5.69E-01 |
| 32 | rs7444 | 0.955(0.931-0.980) | 5.03E-04 | 0.965(0.932-1.000) | 4.97E-02 | 1.012(0.948-1.081) | 7.15E-01 |
| 33 | rs7726414 | 0.954(0.930-0.979) | 3.19E-04 | 0.962(0.930-0.995) | 2.61E-02 | 1.018(0.953-1.086) | 6.02E-01 |
| 34 | rs7941765 | 0.955(0.931-0.980) | 4.91E-04 | 0.963(0.931-0.996) | 2.99E-02 | 1.017(0.953-1.086) | 6.12E-01 |
| 35 | rs849142 | 0.955(0.931-0.980) | 4.36E-04 | 0.963(0.931-0.997) | 3.42E-02 | 1.010(0.947-1.079) | 7.56E-01 |
| 36 | rs9311676 | 0.956(0.931-0.981) | 5.86E-04 | 0.964(0.931-0.998) | 3.96E-02 | 1.016(0.952-1.084) | 6.38E-01 |
| 37 | rs9462027 | 0.955(0.931-0.980) | 5.40E-04 | 0.965(0.932-0.999) | 4.19E-02 | 1.009(0.945-1.077) | 7.95E-01 |
| 38 | rs9652601 | 0.954(0.930-0.978) | 2.58E-04 | 0.961(0.930-0.994) | 2.15E-02 | 1.015(0.950-1.083) | 6.66E-01 |
| 39 | rs9782955 | 0.955(0.931-0.980) | 4.80E-04 | 0.964(0.931-0.998) | 3.95E-02 | 1.015(0.951-1.083) | 6.57E-01 |
|  | All | 0.956(0.932-0.981) | 5.33E-04 | 0.965(0.933-0.999) | 4.35E-02 | 1.013(0.949-1.081) | 6.93E-01 |

IVW MR: Odd ratio (OR) and 95% confidence interval (CI) for, overall endometrial cancer (OR_1_), endometrioid endometrial cancer (OR_2_), and non-endometrioid endometrial cancer (OR3) and Hip and/or knee OA (OR4); P value for, overall endometrial cancer (P_1_), endometrioid endometrial cancer (P_2_), and non-endometrioid endometrial cancer (P3).

**Table S5.** Univariable MR results of the causal effect of SLE on endometrial cancer by manually pruning pleiotropic variants

| **Outcome** | **IVs** | **OR** | **95% CI lower** | **95% CI upper** | **P** | **Method** |
| --- | --- | --- | --- | --- | --- | --- |
| Overall endometrial cancer | 36 | 0.952 | 0.928 | 0.978 | <0.001 | IVW |
|  | 36 | 0.954 | 0.919 | 0.990 | 0.012 | Weighted median |
|  | 36 | 0.931 | 0.885 | 0.978 | 0.008 | Weighted mode |
|  | 36 | 0.928 | 0.883 | 0.976 | 0.006 | MR-Egger |
| Endometrioid endometrial cancer | 36 | 0.962 | 0.928 | 0.997 | 0.033 | IVW |
|  | 36 | 0.944 | 0.902 | 0.987 | 0.012 | Weighted median |
|  | 36 | 0.913 | 0.859 | 0.971 | 0.006 | Weighted mode |
|  | 36 | 0.935 | 0.873 | 1.001 | 0.060 | MR-Egger |
| Non-endometrioid endometrial cancer | 36 | 1.002 | 0.938 | 1.071 | 0.949 | IVW |
|  | 36 | 1.010 | 0.917 | 1.112 | 0.841 | Weighted median |
|  | 36 | 1.030 | 0.929 | 1.142 | 0.587 | Weighted mode |
|  | 36 | 1.051 | 0.928 | 1.191 | 0.440 | MR-Egger |

Three (rs9462027, rs849142 and rs2736340) out of the 39 IVs were associated with body mass index (*P* < 1×10^-5^), and were therefor excluded.


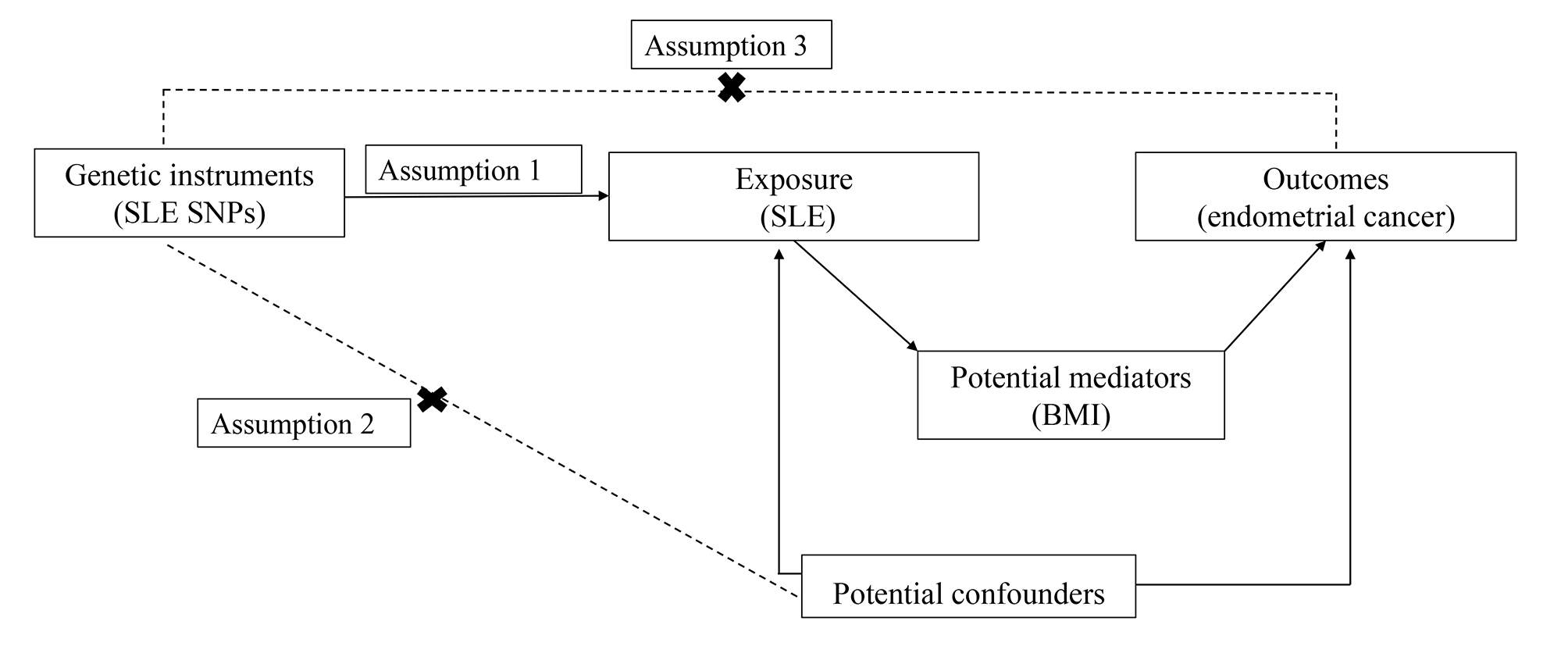


**Figure S1.** Study design of the current study: Mendelian randomization analysis. The MR approach relies on the following three assumptions: assumption 1, the genetic variants must be strongly correlated with the exposure (here, SLE); assumption 2, the genetic variants must be unrelated to any confounding factors that are associated with the outcome (here, endometrial cancer); and assumption 3, the genetic variants must affect the outcome only through exposure factors rather than via alternative ways.

**
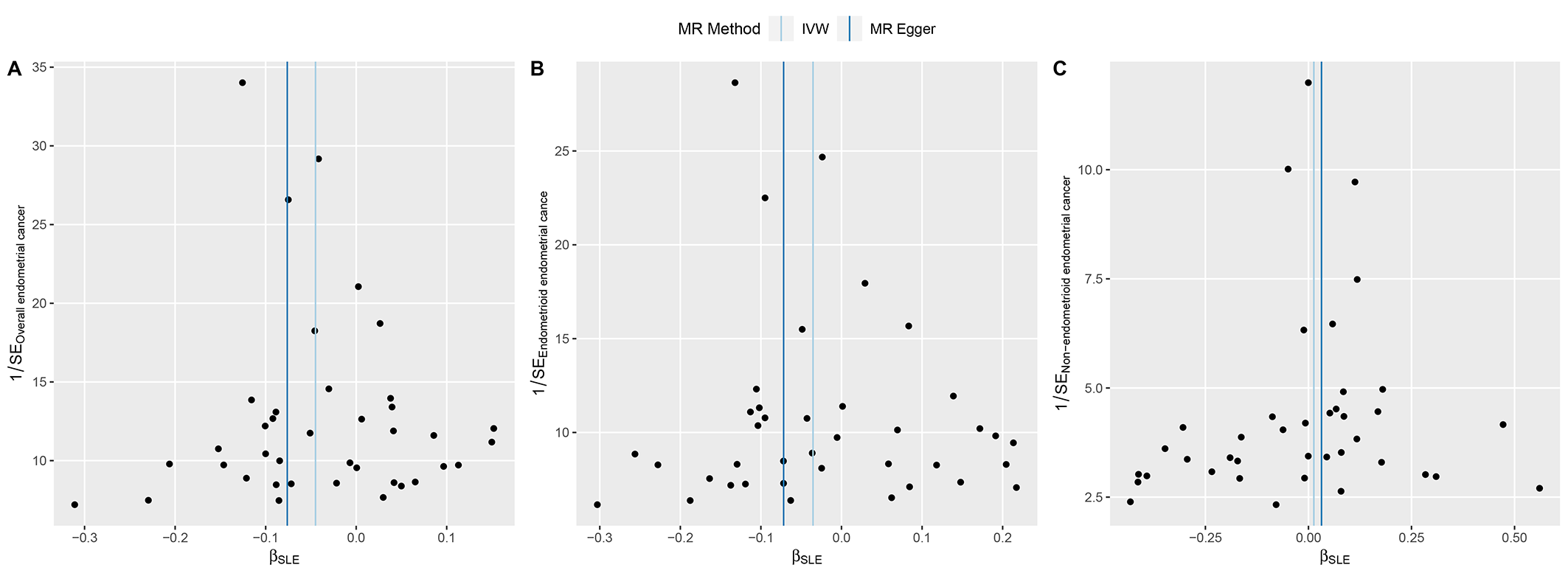
**

**Figure S2.** Funnel plots to show symmetrical distribution of individual variant estimates around the point estimate

**
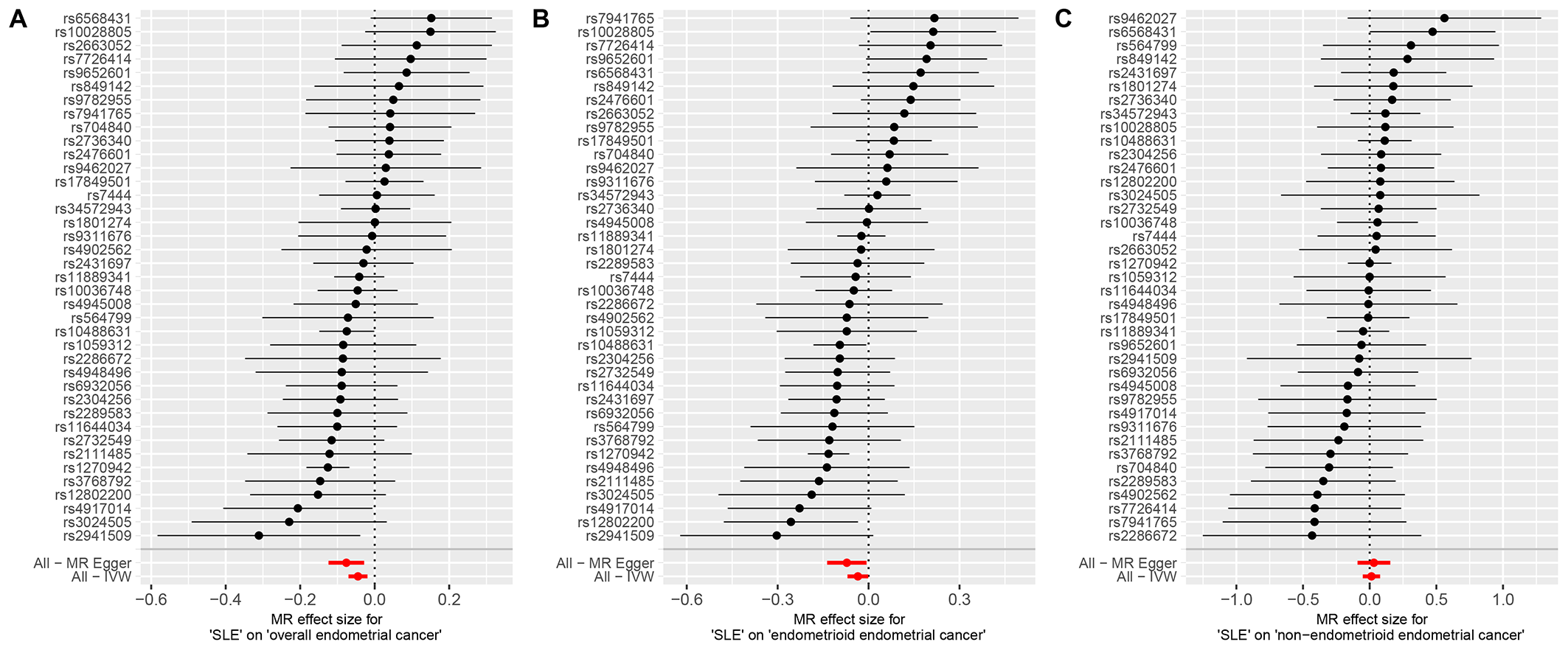
Figure S3.** Forest plot individual and combined SNP MR-estimated effects sizes for SLE on endometrial cancer
